# Supplementary material for: Evaluating batch correction methods for image-based cell profiling
Source: Nat Commun. 2024 Aug 2;15:6516. doi: 10.1038/s41467-024-50613-5 (PMC11297288; doi:10.1038/s41467-024-50613-5)
Supplement: Supplementary file 1 — Supplementary material [file 41467_2024_50613_MOESM1_ESM.pdf]

Evaluating batch correction methods for image-based cell profiling

John Arevalo Ellen Su Jessica D. Ewald Robert van Dijk Anne E. Carpenter
Shantanu Singh

Supplementary Material

Preprocessing exploration

We explored combinations of the four steps above along with strategies to deal with outliers such as imputation (with KNN and median), clipping, and feature dropping. We choose the most convenient pipeline based on the mAP scores in Scenario 1.

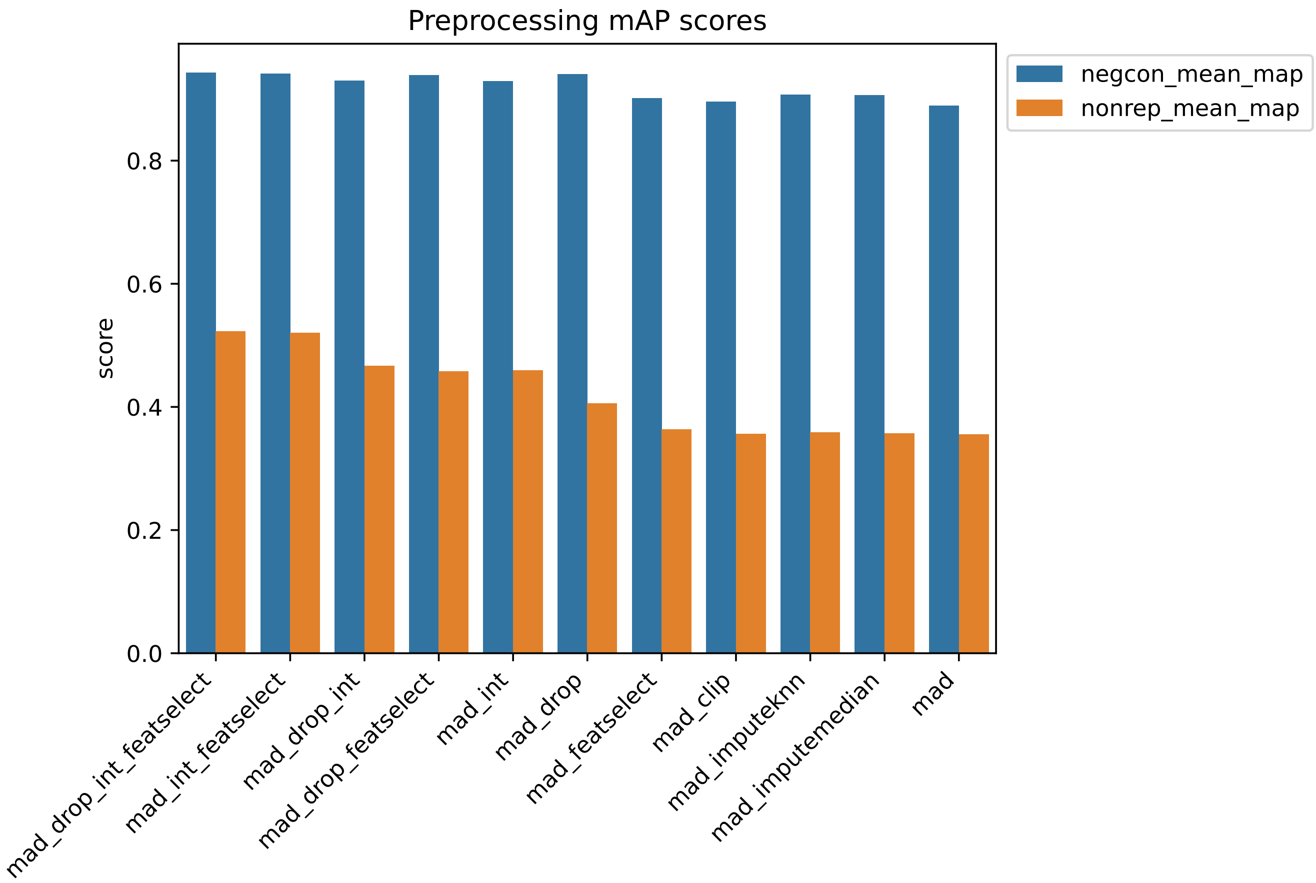

Supplementary Figure 1: preprocessing mAP scores in Scenario 1 (n=8,064 wells). Every combination is encoded in the name as follows. mad: median absolute deviation normalization; clip: clip outlier values to 500; drop: drop any column with an outlier value; imputemedian: impute outliers with median value; imputeknn: impute outlier values with KNN; featselect: Feature selection process using variance_threshold, correlation_threshold operations from PyCytominer [1]; int: rank-based Inverse normal transformation. negcon and nonrep represent scores for replicability [2]. Source data are provided as a Source Data file.

Scenario 1

A

Method	Batch correction: Graph connectivity	Batch correction: KBET	Batch correction: LISI batch	Batch correction: Silhouette batch	Bio metrics: LISI label	Bio metrics: Leiden ARI	Bio metrics: Leiden NMI	Bio metrics: Silhouette label	Bio metrics: mAP (controls)	Bio metrics: mAP (nonrep)	Aggregate score: Batch correction	Aggregate score: Bio metrics	Aggregate score: Overall
**fastMNN**	0.81	0.46	0.44	0.76	0.98	0.05	0.58	0.55	0.95	0.63	0.62	0.62	0.62
**Seurat RPCA**	0.81	0.44	0.43	0.78	0.98	0.06	0.60	0.53	0.95	0.60	0.62	0.62	0.62
**Seurat CCA**	0.80	0.42	0.46	0.77	0.98	0.07	0.59	0.52	0.95	0.61	0.61	0.62	0.62
**Harmony**	0.79	0.42	0.40	0.74	0.98	0.07	0.60	0.55	0.95	0.60	0.59	0.62	0.61
**Combat**	0.78	0.44	0.41	0.84	0.98	0.05	0.56	0.53	0.94	0.55	0.62	0.60	0.61
**MNN**	0.78	0.44	0.40	0.84	0.98	0.07	0.56	0.52	0.94	0.54	0.61	0.60	0.61
**DESC**	0.78	0.39	0.42	0.74	0.98	0.06	0.58	0.55	0.95	0.56	0.58	0.61	0.60
**Baseline**	0.77	0.41	0.39	0.85	0.98	0.06	0.55	0.52	0.94	0.52	0.61	0.60	0.60
**Sphering**	0.74	0.39	0.37	0.87	0.98	0.06	0.54	0.52	0.94	0.51	0.59	0.59	0.59
**Scanorama**	0.54	0.72	0.53	0.59	0.98	0.09	0.56	0.45	0.94	0.49	0.59	0.59	0.59
**scVI**	0.72	0.39	0.43	0.70	0.98	0.06	0.58	0.53	0.94	0.56	0.56	0.61	0.59

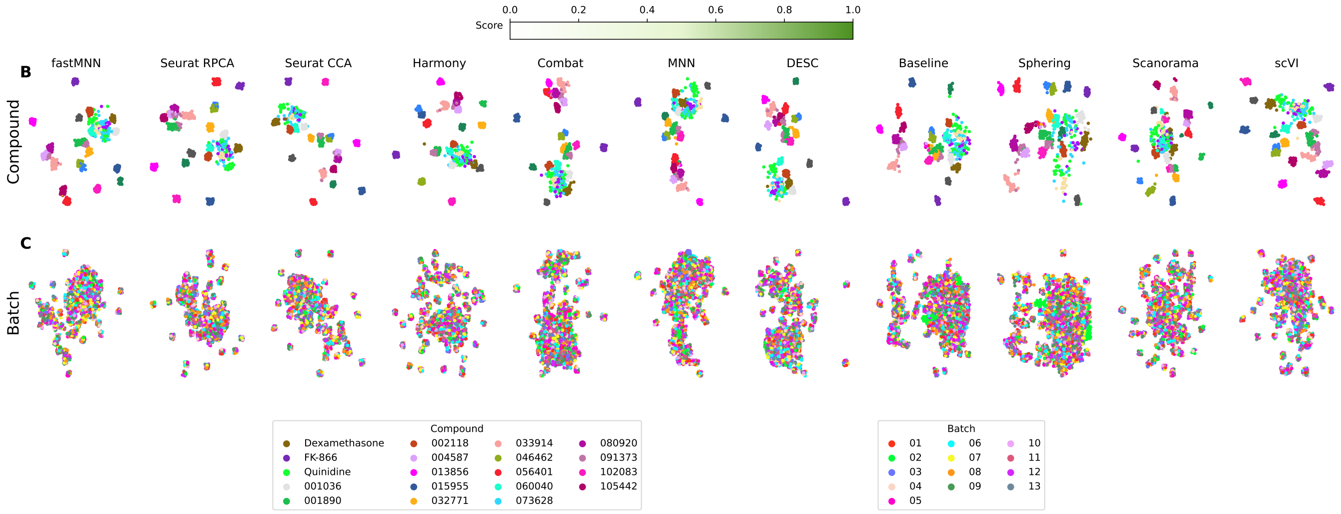

Supplementary Figure 2: Evaluation Scenario 1. A) Quantitative comparison of ten batch correction methods measuring batch effect removal (four batch correction metrics) and conservation of biological variance (six bio-metrics). Metrics are mean aggregated by category. Overall score is the weighted sum of aggregated batch correction and bio-metrics with 0.4 and 0.6 weights respectively. Visualization of integrated data colored by B) Compound, and C) Batch. Left-to-right layout reflects the methods' descending order of performance. We selected 18 out of 302 compounds with replicates in different well positions to account for position effects that may cause profiles to look similar. Alphanumeric IDs denote positive controls. Source data are provided as a Source Data file.

Scenario 2

A

Method	Batch correction: Graph connectivity	Batch correction: KBET	Batch correction: LISI batch	Batch correction: Silhouette batch	Bio metrics: LISI label	Bio metrics: Leiden ARI	Bio metrics: Leiden NMI	Bio metrics: Silhouette label	Bio metrics: mAP (controls)	Bio metrics: mAP (nonrep)	Aggregate score: Batch correction	Aggregate score: Bio metrics	Aggregate score: Overall
**Scanorama**	0.53	0.66	0.67	0.79	0.98	0.04	0.42	0.47	0.92	0.46	0.66	0.55	0.59
**Seurat CCA**	0.64	0.28	0.55	0.86	0.98	0.04	0.45	0.49	0.93	0.47	0.58	0.56	0.57
**Harmony**	0.64	0.26	0.55	0.86	0.98	0.04	0.42	0.48	0.92	0.46	0.58	0.55	0.56
**Seurat RPCA**	0.65	0.24	0.46	0.84	0.98	0.05	0.46	0.49	0.93	0.47	0.55	0.56	0.56
**fastMNN**	0.56	0.27	0.52	0.80	0.97	0.04	0.39	0.45	0.92	0.45	0.54	0.54	0.54
**scVI**	0.59	0.16	0.48	0.74	0.98	0.04	0.45	0.47	0.90	0.47	0.49	0.55	0.53
**MNN**	0.62	0.10	0.19	0.76	0.98	0.04	0.42	0.48	0.90	0.42	0.42	0.54	0.49
**Combat**	0.62	0.05	0.16	0.76	0.98	0.03	0.42	0.48	0.91	0.42	0.40	0.54	0.48
**DESC**	0.59	0.11	0.32	0.67	0.97	0.03	0.38	0.42	0.89	0.42	0.42	0.52	0.48
**Baseline**	0.60	0.04	0.17	0.75	0.98	0.04	0.42	0.48	0.90	0.41	0.39	0.54	0.48
**Sphering**	0.58	0.03	0.13	0.76	0.98	0.03	0.40	0.48	0.91	0.39	0.38	0.53	0.47

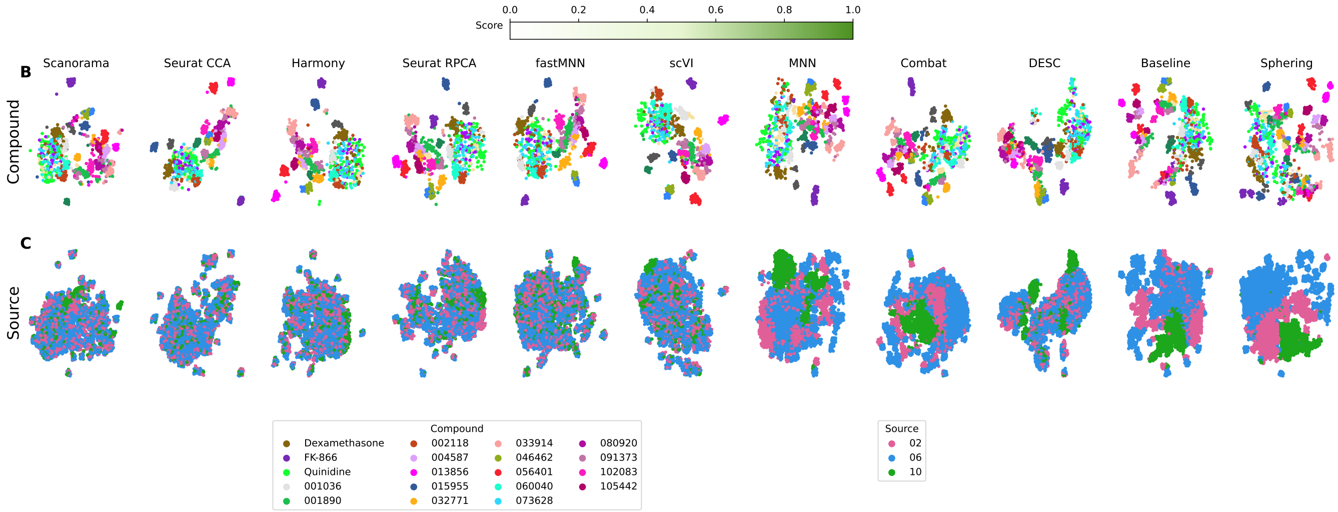

Supplementary Figure 3: Evaluation Scenario 2. A) Quantitative comparison of ten batch correction methods measuring batch effect removal (four batch correction metrics) and conservation of biological variance (six bio-metrics). Metrics are mean aggregated by category. Overall score is the weighted sum of aggregated batch correction and bio-metrics with 0.4 and 0.6 weights respectively. Visualization of integrated data colored by B) Compound, and C) Source. Left-to-right layout reflects the methods' descending order of performance. We selected 18 out of 302 compounds with replicates in different well positions to account for position effects that may cause profiles to look similar. Alphanumeric IDs denote positive controls. Source data are provided as a Source Data file.

Scenario 3

A

Method	Batch correction: Graph connectivity	Batch correction: KBET	Batch correction: LISI batch	Batch correction: Silhouette batch	Bio metrics: LISI label	Bio metrics: Leiden ARI	Bio metrics: Leiden NMI	Bio metrics: Silhouette label	Bio metrics: mAP (controls)	Bio metrics: mAP (nonrep)	Aggregate score: Batch correction	Aggregate score: Bio metrics	Aggregate score: Overall
**Seurat CCA**	0.55	0.17	0.51	0.79	1.00	0.02	0.30	0.29	0.28	0.06	0.51	0.32	0.40
**Scanorama**	0.54	0.33	0.36	0.76	1.00	0.01	0.29	0.27	0.20	0.05	0.50	0.30	0.38
**fastMNN**	0.54	0.14	0.48	0.72	1.00	0.02	0.30	0.21	0.24	0.06	0.47	0.31	0.37
**Seurat RPCA**	0.55	0.15	0.28	0.79	1.00	0.02	0.32	0.30	0.25	0.05	0.44	0.32	0.37
**Harmony**	0.54	0.06	0.25	0.79	1.00	0.02	0.32	0.31	0.24	0.05	0.41	0.32	0.36
**scVI**	0.54	0.07	0.27	0.74	1.00	0.02	0.32	0.23	0.24	0.05	0.40	0.31	0.35
**Sphering**	0.54	0.02	0.00	0.80	1.00	0.00	0.26	0.35	0.21	0.03	0.34	0.31	0.32
**Combat**	0.54	0.03	0.02	0.76	1.00	0.01	0.29	0.30	0.22	0.04	0.34	0.31	0.32
**MNN**	0.54	0.03	0.01	0.75	1.00	0.01	0.29	0.29	0.20	0.04	0.33	0.30	0.32
**Baseline**	0.54	0.03	0.00	0.74	1.00	0.00	0.28	0.29	0.19	0.03	0.33	0.30	0.31
**DESC**	0.53	0.00	0.15	0.43	1.00	0.02	0.38	0.04	0.14	0.02	0.28	0.27	0.27

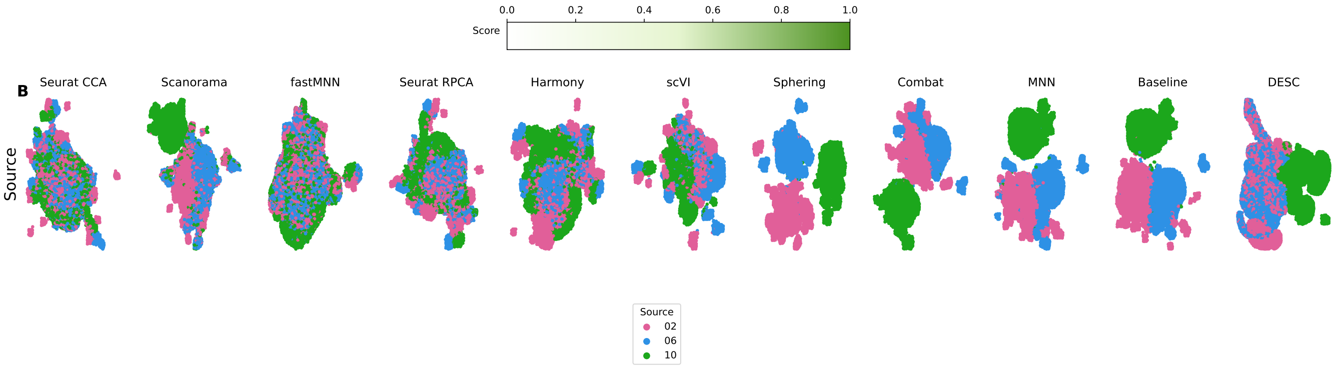

Supplementary Figure 4: Evaluation Scenario 3. A) Quantitative comparison of ten batch correction methods measuring batch effect removal (four batch correction metrics) and conservation of biological variance (six bio-metrics). Metrics are mean aggregated by category. Overall score is the weighted sum of aggregated batch correction and bio-metrics with 0.4 and 0.6 weights respectively. B) Visualization of integrated data colored by Source. Left-to-right layout reflects the methods' descending order of performance. Source data are provided as a Source Data file.

Scenario 5

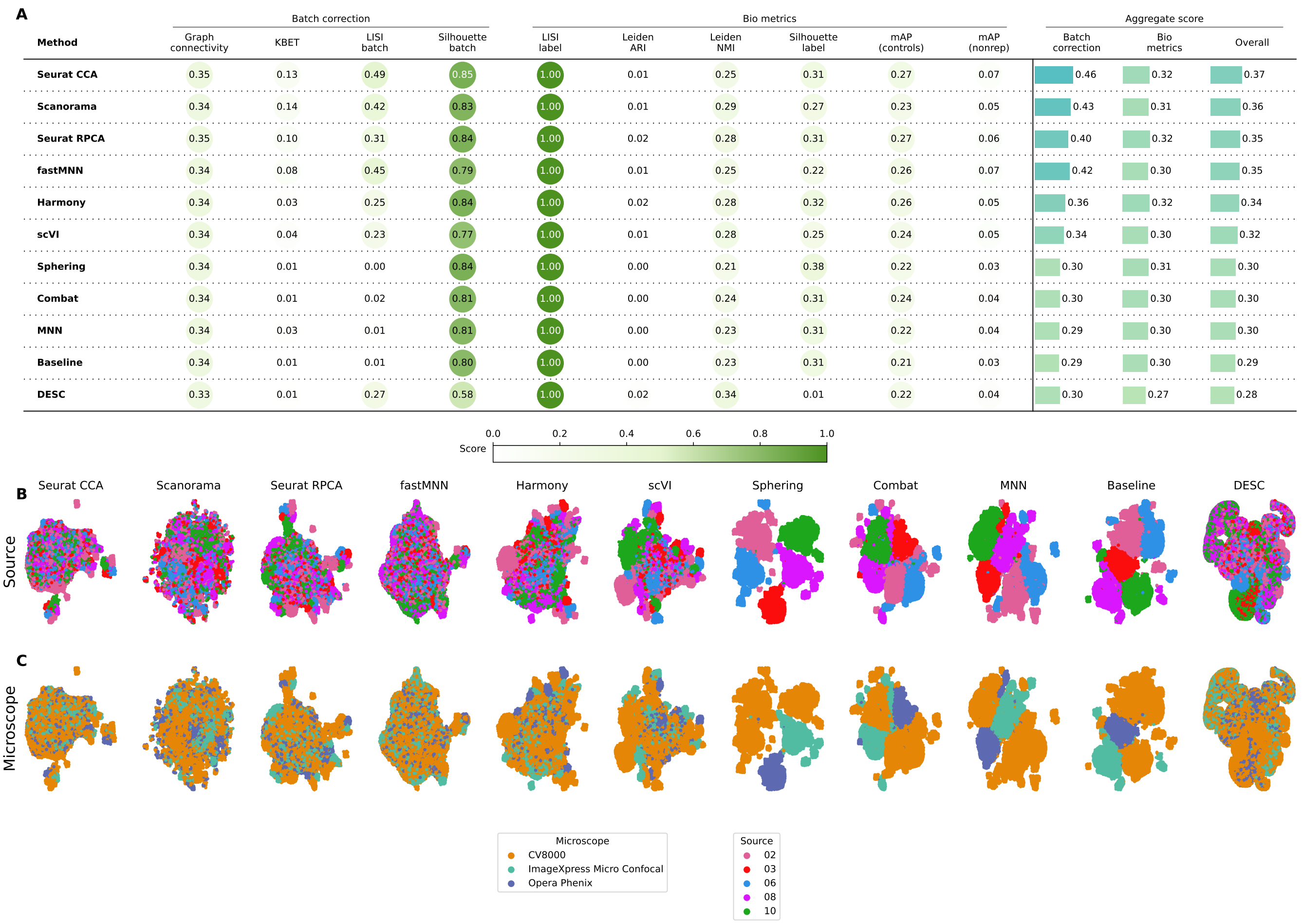

Method	Batch correction: Graph connectivity	KBET	LISI batch	Silhouette batch	Bio metrics: LISI label	Leiden ARI	Leiden NMI	Silhouette label	mAP (controls)	mAP (nonrep)	Aggregate score: Batch correction	Bio metrics	Overall
Seurat CCA	0.35	0.13	0.49	0.85	1.00	0.01	0.25	0.31	0.27	0.07	0.46	0.32	0.37
Scanorama	0.34	0.14	0.42	0.83	1.00	0.01	0.29	0.27	0.23	0.05	0.43	0.31	0.36
Seurat RPCA	0.35	0.10	0.31	0.84	1.00	0.02	0.28	0.31	0.27	0.06	0.40	0.32	0.35
fastMNN	0.34	0.08	0.45	0.79	1.00	0.01	0.25	0.22	0.26	0.07	0.42	0.30	0.35
Harmony	0.34	0.03	0.25	0.84	1.00	0.02	0.28	0.32	0.26	0.05	0.36	0.32	0.34
scVI	0.34	0.04	0.23	0.77	1.00	0.01	0.28	0.25	0.24	0.05	0.34	0.30	0.32
Sphering	0.34	0.01	0.00	0.84	1.00	0.00	0.21	0.38	0.22	0.03	0.30	0.31	0.30
Combat	0.34	0.01	0.02	0.81	1.00	0.00	0.24	0.31	0.24	0.04	0.30	0.30	0.30
MNN	0.34	0.03	0.01	0.81	1.00	0.00	0.23	0.31	0.22	0.04	0.29	0.30	0.30
Baseline	0.34	0.01	0.01	0.80	1.00	0.00	0.23	0.31	0.21	0.03	0.29	0.30	0.29
DESC	0.33	0.01	0.27	0.58	1.00	0.02	0.34	0.01	0.22	0.04	0.30	0.27	0.28

Supplementary Figure 5: Evaluation Scenario 5. A) Quantitative comparison of ten batch correction methods measuring batch effect removal (four batch correction metrics) and conservation of biological variance (six bio-metrics). Metrics are mean aggregated by category. Overall score is the weighted sum of aggregated batch correction and bio-metrics with 0.4 and 0.6 weights respectively. Visualization of integrated data colored by B) Source, and C) Microscope. Left-to-right layout reflects the methods' descending order of performance. Source data are provided as a Source Data file.

Isolated compounds performance

Around 30% of the compounds of Scenario 3 are present in all three sources (sources 2, 6, and 10). We used this scenario to assess the replicate retrieval performance of sub-populations of compounds that are not shared between different batches (i.e. sources, in this setup). We used the corrected profiles from the best-performing correction method in the scenario – Seurat CCA– to evaluate. We picked the 10,136 compounds that present in sources 2 and 6 but not in source 10 (i.e., they are isolated to sources 2 and 6). We compared the performance of this subpopulation (named as two sources in Sup Figure 6) with the performance of a subpopulation of 23,782 compounds present in all of the three sources (named as three sources in Sup Figure F). Then we compute the mAP (control) score for each subpopulation, noting that we pick only the replicates from source 2 and source 6 and ignoring the replicate from source 10. We observed that the compounds that exclusively belong to two sources performed better than compounds present in all three sources, which contradicts the over-correction hypothesis. A likely explanation is that the correction task gets more difficult as there are more sources to align.

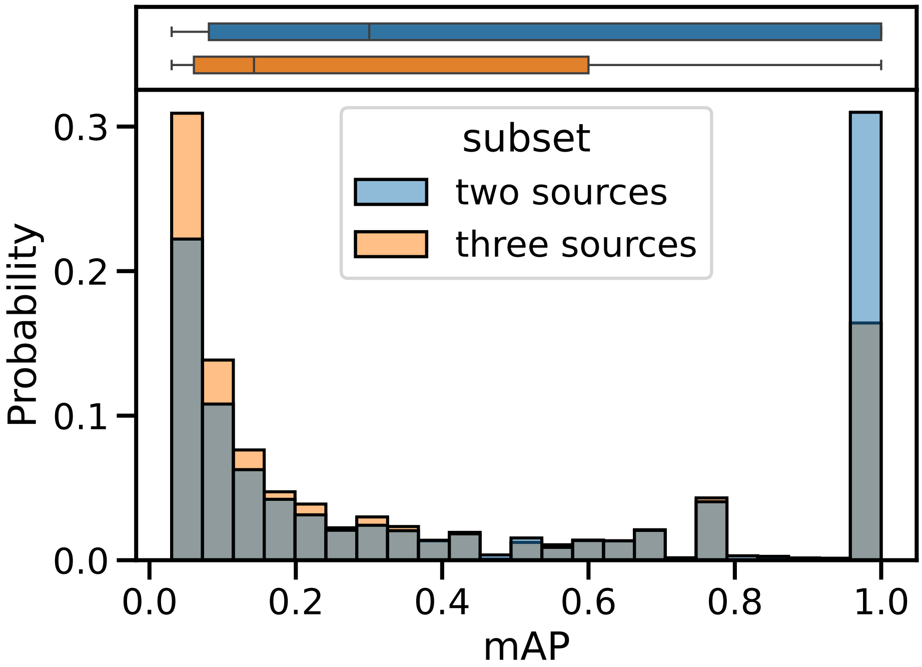

Supplementary Figure 6: Comparison of the replicate retrieval performance (mAP) of sub-populations of compounds that are not shared between different batches. The sub-population present in only two sources (n=10,136 compounds) performed better than the sub-population in three sources (n=23,782 compounds). Data extracted from the Scenario 3. Source data are provided as a Source Data file.

Runtime analysis

We measured the runtime for non-GPU methods and metrics across the five scenarios using a c6i.16xlarge AWS EC2 instance equipped with 64 cores and 128GB of RAM. A log-log plot of the results (Sup Figure 7) suggests a power-law relationship between runtime and sample size. Extrapolating this trend reveals that applying Harmony, one of the top-performing methods, at the single-cell level (Sup Table 1) would be prohibitively time-consuming. Processing a single plate would take approximately 2.6 hours, a single batch would require 33 hours, and a single source (out of 13 in the full JUMP Cell Painting dataset) would take 11 days. Moreover, loading a single source would require 2.7 TB of memory. Similarly, the runtime extrapolation of Seurat CCA estimates that processing the entire JUMP dataset at the well level, containing approximately 890,000 well-level profiles, would take 17 days.

Level	Mean	Median
**Per Well**	1,846	1,520
**Per Plate**	708,819	575,946
**Per Batch**	14,129,906	11,292,977
**Per Source**	172,384,854	147,953,548
**Total (sources 2,3,6,8,10)**	861,924,272	
**Total (all 13 JUMP sources)**	1,834,731,584	

Supplementary Table 1: Count of single cells at different levels in the JUMP CP Dataset.

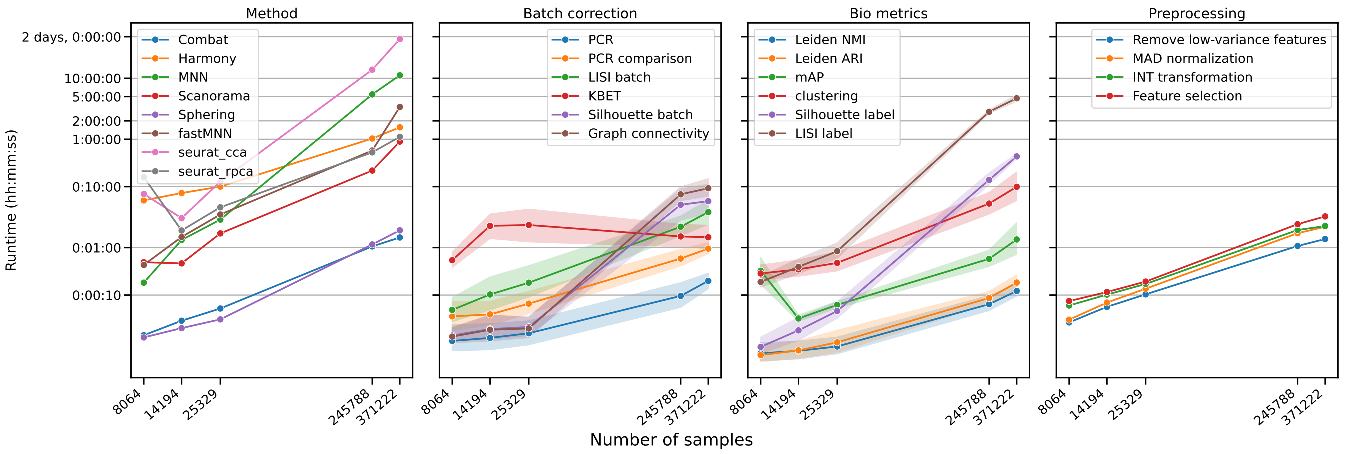

Supplementary Figure 7: Runtime analysis of methods, metrics, and preprocessing steps for all the scenarios. Scenario 1, 2, and 4 (first three ticks in the x-axis) have JUMP-Target-2-Compound plates only with 300 unique compounds. Scenarios 3 and 5 (last two ticks in the x-axis) have Production plates with 80,000 unique compounds. Both axes are log-scaled. kBET runtime trend is constant because it only evaluates compounds with more than 15 replicates. The Clustering step is required for LISI, NMI, ARI, Graph connectivity, and kBET. Source data are provided as a Source Data file.

Performance distribution

Method	Batch correction	Bio metrics	Overall	Batch correction#	Bio metrics #	Overall #
seurat_cca	0.54	0.48	0.50	1	2	1
seurat_rpca	0.50	0.48	0.49	3.5	1	2
harmony	0.49	0.47	0.48	5	3	3
scanorama	0.55	0.46	0.49	2	7	4
fastMNN	0.51	0.46	0.48	3.5	5	5
scvi	0.45	0.46	0.46	6.5	4	6
combat	0.40	0.46	0.44	6.5	6	7
mnn	0.41	0.45	0.44	8	8	8
sphering	0.39	0.45	0.43	9	9	9
baseline	0.40	0.45	0.43	10.5	10	10
desc	0.37	0.43	0.40	10.5	11	11

Supplementary Table 2: Comparison of batch correction methods by aggregating scores across scenarios. Metrics are mean-aggregated by category. Overall score is the weighted sum of aggregated batch correction and bio-metrics with 0.4 and 0.6 weights respectively. The ranks of these scores are in the corresponding columns indicated with #. Methods are sorted by overall rank.

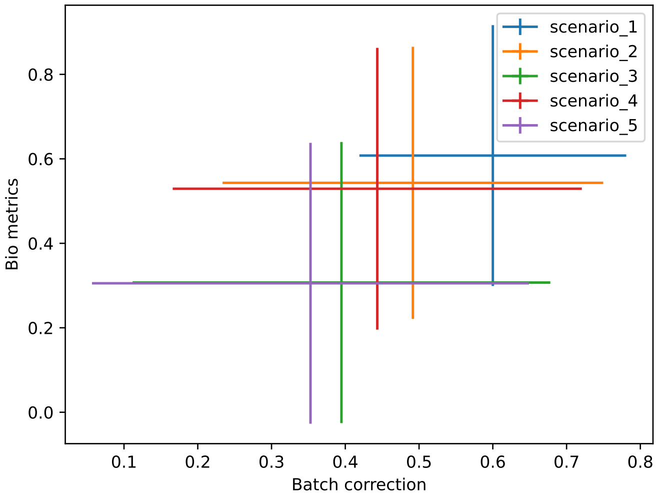

Supplementary Figure 8: Scatter plot of the mean Batch correction and Bio-metrics for all the tested methods across the five scenarios, reflecting the increasing difficulty of scenarios. Bars represent one standard deviation in the respective axis. Source data are provided as a Source Data file.

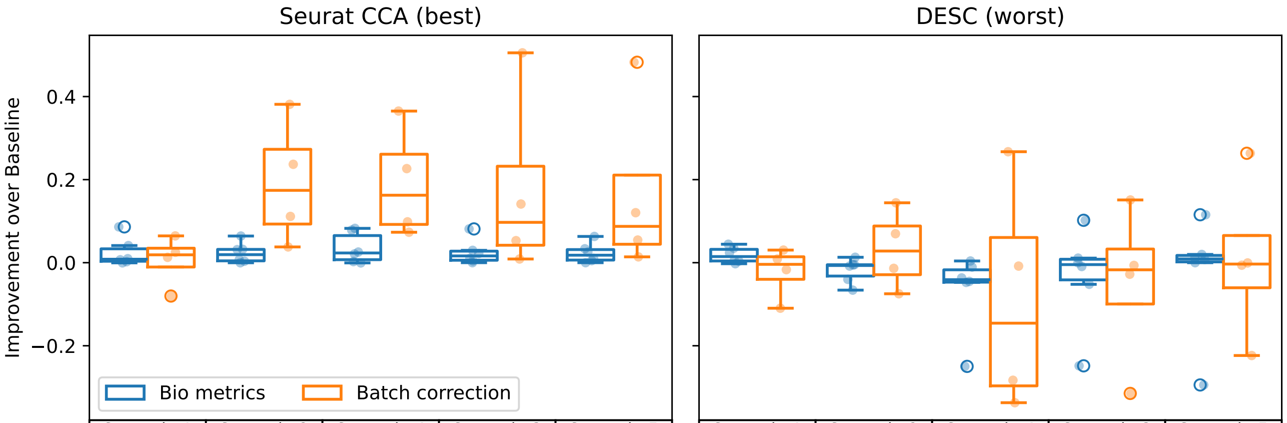

	Seurat CCA (best)					DESC (worst)				
	Scenario 1	Scenario 2	Scenario 4	Scenario 3	Scenario 5	Scenario 1	Scenario 2	Scenario 4	Scenario 3	Scenario 5
Sources	1	3	5	3	5	1	3	5	3	5
Microscopes	1	1	3	1	3	1	1	3	1	3
Compounds	306	306	306	80000+	80000+	306	306	306	80000+	80000+

Supplementary Figure 9: Comparison of best and worst batch correction methods, reflecting the variability of the performance with respect to the complexity of the scenarios (scenarios are sorted by overall mean score). Source data are provided as a Source Data file.

Scenario	Batch correction			Bio metrics		
	**Graph connectivity**	**kBET**	**LISI batch**	**LISI label**	**Leiden ARI**	**Leiden NMI**
**Scenario 1**	0.594 (10)	0.229 (11)	0.344 (11)	0.982 (-)	0.042 (11)	0.334 (11)
**Scenario 2**	0.255 (11)	0.316 (2)	0.811 (4)	0.975 (-)	0.028 (11)	0.247 (11)
**Scenario 3**	0.537 (10)	0.065 (6)	0.544 (1)	1.000 (-)	0.017 (6)	0.234 (11)
**Scenario 4**	0.262 (10)	0.658 (2)	0.642 (1)	0.978 (-)	0.030 (5)	0.180 (11)
**Scenario 5**	0.336 (10)	0.012 (8)	0.006 (8)	1.000 (-)	0.002 (-)	0.229 (10)

Supplementary Table 3: Evaluation metrics for BBKNN [3] on the benchmark datasets. While BBKNN was considered for inclusion in the study based on its batch correction capabilities via k-NN graph construction [4, 5] it was ultimately excluded from the main analysis. The primary reason for this exclusion is that BBKNN does not correct the underlying profiles, limiting both the applicable evaluation metrics and downstream analyses that rely on corrected vectors or distances beyond the k-NN graph. Metrics were computed for BBKNN whenever possible and are reported here for completeness.

Implementation notes

- For scVI, we shifted the data to the feasible space. (i.e. transform each feature $x_i = x_i - \min(x) + 1$.
- The nature of the image-based profile data involving low number of replicates and high number of compounds limits kBET, which relies on a higher (> 15) number of samples per biological concept.
- mAP is the only metric able to capture the performance of the models when there are as few as only two replicates of a compound.
- We optimize the preprocessing pipeline based on a mAP.
- We adjust DESC convergence hyperparameters to avoid collapsed representations (default parameters converged to vectors with only -1, 1 values (output from a tanh activation))
- We increase the number of Harmony clusters from 50 to 300 and iterations from 10 to 20.
- We increase the number of latent dimensions in scVI from 10 to 30.
- Combat implementation from scanpy has no hyperparameters.
- We use the default hyperparameters for MNN (neighbor size=20), Scanorama (KNN=20, alpha=0.1, sigma=15) and scVI (num_units=128, dropout=0.1).

Supplementary References

1. Serrano, E. *et al.* Reproducible image-based profiling with Pycytominer. en (Nov. 2023).
2. Kalinin, A. A. *et al.* A versatile information retrieval framework for evaluating profile strength and similarity. *bioRxiv* (2024).
3. Polański, K. *et al.* BBKNN: fast batch alignment of single cell transcriptomes. en. *Bioinformatics* **36,** 964–965 (Feb. 2020).
4. Chen, W. *et al.* A multicenter study benchmarking single-cell RNA sequencing technologies using reference samples. en. *Nat. Biotechnol.* **39,** 1103–1114 (Sept. 2021).
5. Luecken, M. D. *et al.* Benchmarking atlas-level data integration in single-cell genomics. en. *Nat. Methods* **19,** 41–50 (Jan. 2022).
